# Potential Roles of Dental Pulp Stem Cells in Neural Regeneration and Repair

**DOI:** 10.1155/2018/1731289

**Published:** 2018-05-07

**Authors:** Lihua Luo, Yan He, Xiaoyan Wang, Brian Key, Bae Hoon Lee, Huaqiong Li, Qingsong Ye

**Affiliations:** ^1^WMU-UQ Group for Regenerative Medicine, Institute of Stem Cells and Tissue Engineering, School of Stomatology, Wenzhou Medical University, Wenzhou 325035, China; ^2^School of Dentistry, The University of Queensland, Herston, QLD 4006, Australia; ^3^School of Biomedical Sciences, The University of Queensland, Brisbane, QLD 4072, Australia; ^4^School of Biomedical Engineering, School of Ophthalmology & Optometry and Eye Hospital, Wenzhou Medical University, Wenzhou 325035, China; ^5^Wenzhou Institute of Biomaterials and Engineering, CAS, Wenzhou 325011, China; ^6^Engineering Research Center of Clinical Functional Materials and Diagnosis & Treatment Devices of Zhejiang Province, Wenzhou Institute of Biomaterials and Engineering, CAS, Wenzhou 325011, China

## Abstract

This review summarizes current advances in dental pulp stem cells (DPSCs) and their potential applications in the nervous diseases. Injured adult mammalian nervous system has a limited regenerative capacity due to an insufficient pool of precursor cells in both central and peripheral nervous systems. Nerve growth is also constrained by inhibitory factors (associated with central myelin) and barrier tissues (glial scarring). Stem cells, possessing the capacity of self-renewal and multicellular differentiation, promise new therapeutic strategies for overcoming these impediments to neural regeneration. Dental pulp stem cells (DPSCs) derive from a cranial neural crest lineage, retain a remarkable potential for neuronal differentiation, and additionally express multiple factors that are suitable for neuronal and axonal regeneration. DPSCs can also express immunomodulatory factors that stimulate formation of blood vessels and enhance regeneration and repair of injured nerve. These unique properties together with their ready accessibility make DPSCs an attractive cell source for tissue engineering in injured and diseased nervous systems. In this review, we interrogate the neuronal differentiation potential as well as the neuroprotective, neurotrophic, angiogenic, and immunomodulatory properties of DPSCs and its application in the injured nervous system. Taken together, DPSCs are an ideal stem cell resource for therapeutic approaches to neural repair and regeneration in nerve diseases.

## 1. Introduction

Traumatic events, iatrogenic injuries, and neurodegenerative diseases can lead to axonal degeneration, inflammation, neuron death, and cytoarchitectural malformation in both the peripheral nervous system (PNS) and central nervous system (CNS) [1–6]. Conventional medical therapies have limited efficacy in supporting functional recovery from nervous damage since the mature nervous system lacks the necessary precursor cells to generate new neurons and glial cells [7]. Recently, stem cell-based strategies in combination with novel technologies (e.g., precisely controlled hydrogels) have heralded potential new therapeutic approaches for addressing nerve regeneration and repair [8–11].

Mesenchymal stem cells (MSCs) harvested from adult tissues are potentially an important therapeutic cell source for treatment of CNS and PNS perturbations since they possess the capacity for both neuronal and glial differentiation. MSCs also express numerous anti-inflammatory and neurotrophic factors supporting nerve repair [8–14]. These multipotent stem cells are present in bone marrow [15, 16], adipose tissue [17, 18], umbilical cord [19, 20], and dental tissue [21–25]. Dental pulp stem cells (DPSCs) can readily be obtained from the third molars, usually discarded as medical waste. DPSCs have MSC-like characteristics such as the ability for self-renewal and multilineage differentiation. These dental pulp-derived MSCs avoid ethical concerns when sourced from other tissue, and they can be obtained without unnecessary invasive procedures, for example, MSCs collected from bone marrow or adipose tissue [9, 26–28]. DPSCs can differentiate into neuron-like cells and secrete neurotrophic factors such as neurotrophin (NT) [29, 30]. In addition, DPSCs express neuron-related markers even before being induced to neuronal differentiation [29, 31, 32]. Taken together, these unique properties make DPSCs an excellent candidate for stem cell-related therapies in nerve diseases.

## 2. Dental Pulp Stem Cells (DPSCs)

### 2.1. The Characteristics of DPSCs

The basic tooth structure consists of an outer enamel layer, a middle dentin layer, and an inner dental pulp layer. It develops from both cranial neural crest-derived mesenchymal stem cells (MSCs) and oral-derived epithelial stem cells in the early stages of embryogenesis [33–35]. Dental pulp, a soft connective tissue containing blood vessels, nerves, and mesenchymal tissue, has a central role in primary and secondary tooth development and ongoing maintenance for instance in reaction to caries [36, 37]. Stem cells can be isolated from the dental pulp tissue and possess MSC-like characteristics including self-renewal and multipotency [21, 38–40]. The first dental pulp-related stem cells were isolated from the third molar dental pulp by Gronthos et al. in 2000 [21]. Subsequently, it was reported that DPSCs could also be isolated from other dental pulps including human exfoliated deciduous teeth [22], human permanent and primary teeth [41], and supernumerary teeth [42]. Meanwhile, they are featured by high-proliferative capacity [43–47]. Most importantly, compared with collection procedures of other tissue-derived stem cells, the collection of DPSCs involves none harm to the donor or invasive surgical procedures [27, 40].

There are currently no specific biomarkers that uniquely define DPSCs. They express MSC-like phenotypic markers such as CD27, CD29, CD44, CD73, CD90, CD105, CD146, CD166, CD271, and STRO-1. Yet they do not express CD34, CD45, CD14, or CD19 and HLA-DR surface molecules [38, 39, 48]. Similar to embryonic stem cells, DPSCs express stemness-related markers such as Oct-4, Nanog, and Sox-2, as well as the cytoskeleton-related markers (Nestin and Vimentin) [29, 49, 50]. In addition, DPSCs express other cranial neural crest cell-related neural markers such as glial fibrillary acidic protein (GFAP), *β*-III tubulin, and microtubule-associated protein-2 (MAP-2) [29, 50, 51].

**Figure 1:**
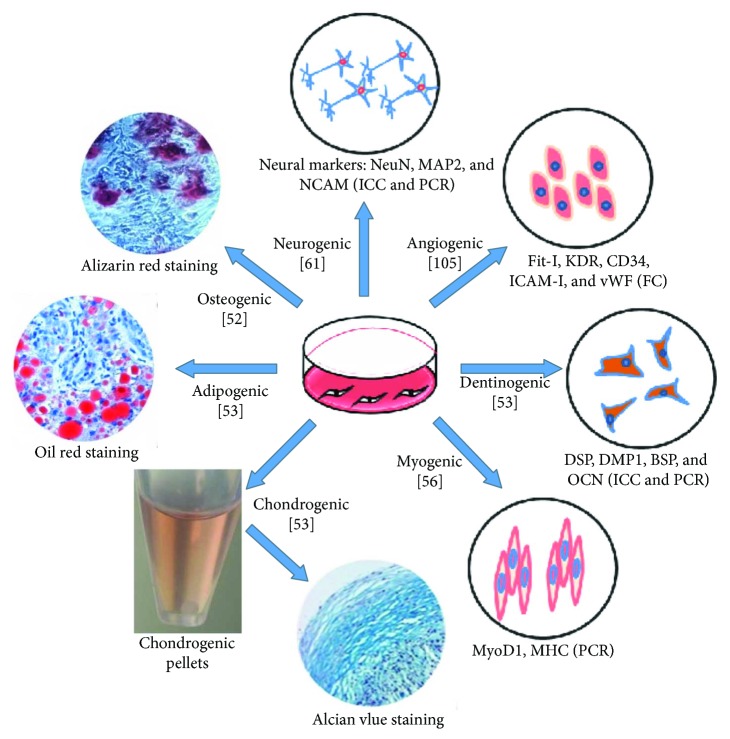
Multidifferentiation potential of DPSCs. DPSCs possess MSC-like properties and are multipotent. *NCAM*: neural cell adhesion molecule; MAP2: microtubule-associated protein 2; NeuN: neuron-specific nuclear protein; Fit-I: VEGF receptor 1; KDR: VEGF receptor 2; CD34: cluster of differentiation 34; ICAM-I: intercellular cell adhesion molecule-1; vWF: von Willebrand factor, DSP: dentin sialoprotein, DMP1: dentin matrix acidic phosphoprotein 1, BSP: bone sialoprotein, OCN: osteocalcin, MyoD1: myoblast determination protein 1; MHC: major histocompatibility complex; PCR: polymerase chain reaction; FC: flow cytometry; ICC: immunocytochemical.

DPSCs are multipotent and can be induced to differentiate into cells for osteogenesis [52], chondrogenesis [53], adipogenesis [53], neurogenesis [54], dentinogenesis [53], odontogenesis [55], and myogenic lineages [56] (Figure 1). Using classic reprogramming factors (e.g., Oct3/4, Sox2, Klf4, and c-MYC), human DPSCs can be converted into induced pluripotent stem cells (iPSCs) [57, 58]. iPSCs exhibit the characteristics of embryonic stem cells and can differentiate into all three germ layers [59, 60]. Human DPSCs have a higher reprogramming efficiency than human dermal fibroblasts because they have a rapid proliferation rate and endogenously express high levels of the reprogramming factors c-MYC and Klf4 [57]. Therefore, DPSCs are potentially an important patient-specific cell source of iPSCs for clinical applications, regenerative medicine, and tissue engineering.

### 2.2. Neuronal Differentiation of DPSCs

DPSCs arise from the cranial neural crest and possess neuron-like characteristics that facilitate their *in vitro* induction into functional neurons. Numerous protocols have been developed to differentiate DPSCs into neurons. Typically, such methods rely on growth factors and various small molecules including basic fibroblast growth factor (bFGF) [61, 62], epidermal growth factor (EGF) [63], nerve growth factor (NGF) [62, 64], brain-derived neurotrophic factor (BDNF) [65], glial cell line-derived neurotrophic factor (GDNF) [66], sonic hedgehog [66], neurotrophin 3 (NT-3) [61], retinoic acid (RA) [63], forskolin [50, 67], and heparin [66] as well as culture supplements such as B27 [61], insulin-transferrin-sodium selenite (ITS) [54], nonessential amino acids [66], and N2 [61, 66]. Under controlled *in vitro* conditions (e.g., spheroid suspension culture in serum-free media), it is possible to differentiate DPSCs into neural lineages that expressed numerous neural markers [61, 63, 64, 68]. Chun et al. have demonstrated that DPSCs could be differentiated into dopaminergic neural cells by the formation of neurosphere [69]. However, huge variations exist in the neural differentiation of DPSCs due to alterations made to the culture of neurosphere, which indicates a delicate regulatory approach is necessary to achieve target differentiation. It is controversial on the timing of neurosphere formation. The study of Gervois et al. showed that it formed in the initial phase during a neural induction [61], whereas studies of Karbanova et al. observed that the neurosphere formed in a rather late phase during the differentiation [70].

Nevertheless, it is possible to bypass neurosphere formation by using endogenous environmental cues and directly differentiate DPSCs into motor and dopaminergic neuronal sublineages [65, 71]. Studies of Chang et al. reported that DPSCs could be directly differentiated into motor neurons by growth factors and small molecules, for example, BDNF and all-trans retinoic acid [71]. Gnanasegaran et al. demonstrated that DPSCs could be induced to differentiate into dopaminergic-like cells by multistage inductive protocols [72]. The study of Singh et al. showed that DPSCs are induced by a two-step method to generate functional dopaminergic neurons: FGF2 first with an addition of BDNF on 9th day. Furthermore, when induced, DPSCs showed much more distinct neuronal characteristics comparing to the other tissue-derived MSCs, for example, bone marrow and adipose tissue [73]. In addition, DPSCs could be differentiated into spiral ganglion neuron-like cells by treating with BDNF, NT-3, and GDNF [74].

**Figure 2:**
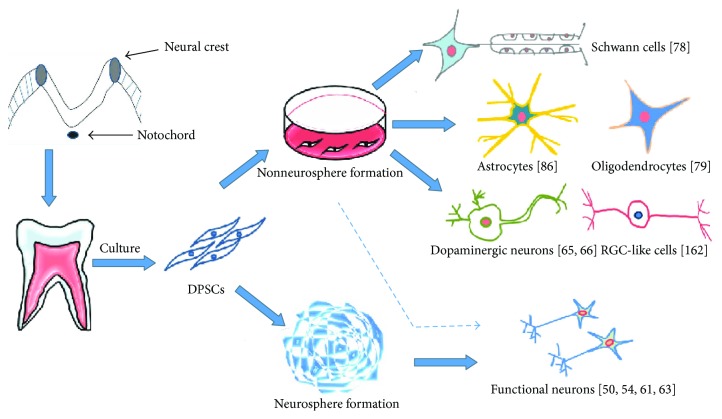
Neural differentiation potential of DPSCs. DPSCs can be induced to differentiate into neural cell lineages including Schwann cells, astrocytes, and dopaminergic neurons.

Typically, a successful neuronal differentiation of DPSCs is confirmed by the increased expression of neuronal markers such as NeuN [61], neurofilament-200 [54], MAP-2 [61, 75], synaptophysin [61], and neural cell adhesion molecules [76]. Few studies have used ultrastructural and/or electrophysiological analyses to confirm the state of differentiation [61]. Previous studies focus on differentiation directions: DPSCs could be differentiated into either neuronal precursor cells (rather than mature neurons capable of generating action potentials) or immature Schwann cells and oligodendrocytes that can support nerve regeneration [77–80] (Figure 2). Recently, research has evolved into in-depth studies on functional and mechanism of DPSC-differentiated neurons. A series of studies have explored the functional activities of DPSC-differentiated neurons in voltage-gated sodium and potassium channels as well the neuronal marker expressions, indicating a successful differentiation is active and functional new neurons have emerged [50, 54, 67]. Further, these predifferentiated DPSCs have been traced and proved well integrated into the central nervous tissue when transplanted in animal models [54, 67]. In summary, versatile differentiations of DPSCs depend on inductive protocols. They can be differentiated into neurons, dopaminergic-like cells, Schwann cells, and oligodendrocytes. Thus, DPSCs are an attractive cell source for stem cell therapy to treat the nervous diseases.

### 2.3. Neuroprotective and Neurotrophic Properties of DPSCs

The efficacy of stem cell therapies in nervous diseases is strongly influenced by trophic factors, for example, BDNF, GDNF, NGF, NT-3, vascular endothelial growth factor (VEGF), and platelet-derived growth factor (PDGF) [29, 30]. The expression of these trophic factors by DPSCs is remarkably higher than those of MSCs derived from bone marrow (BMSCs) and adipose tissue [9, 30]. Further *in vivo* study also demonstrates a more efficient secretion of BDNF and GDNF than BMSCs [81]. These findings confirm that in comparison to other MSCs, DPSCs exhibit superior neuroprotective and neural supportive properties in response to injuries and pathologies of the nervous system. DPSCs have the ability to reduce neurodegeneration in the early stages of neuronal apoptosis and promote motor and sensory neuron survival in spinal cord injury (SCI) by the secretion of BDNF and NGF [82, 83]. Furthermore, trophic factors secreted by DPSCs promoted axon regeneration despite the presence of axon growth inhibitors in the completely transected spinal cord model of SCI [84, 85]. DPSCs also provided both direct and indirect protections against cell death by secreting cytoprotective factors in an ischemic astrocyte model of injury [86, 87]. Compared with other stem cells (DFSCs, SCAP, and BMSCs), DPSCs have shown a higher cytokine expression facilitating neuronal differentiations [88].

### 2.4. Angiogenic Properties of DPSCs

In general, the human body needs abundant nutrition and blood supply in order to maintain its tissues and organs in a healthy condition. The sprouting of new capillaries from existing blood vessels during inflammation and hypoxic conditions depends on the expression and secretion of specific angiogenic trophic factors [89, 90]. Some MSCs are able to promote therapeutic angiogenesis by the secretion of angiogenic growth factors and by differentiating into endothelial cells [91–93]. In particular, DPSCs have been found to secrete and produce abundant angiogenic factors, for example, colony-stimulating factor, interleukin-8, angiogenin, endothelin-1, angiopoietin-1, and insulin-like growth factor binding protein-3 [94–96]. DPSCs also secrete and express other stimulatory growth factors such as VEGF, PDGF, bFGF, and NGF [19, 30, 97]. Synergistically, these factors can promote proliferation and survival of vascular endothelial cells [98, 99] as well as endothelial tubulogenesis [100]. Both the formation and function of new blood vessels are improved by either injection of DPSCs into neuronal disease models or transplantation of DPSCs into ischemia and myocardial infarction animal models [101, 102]. Moreover, Nam et al. observed that by coinjection of DPSCs and HUVECs into immunodeficient mice, microvessel-like structures would be formed, which illustrated that DPSCs could perform as perivascular cells for *in vivo* angiogenesis [103]. DPSCs also have the ability to differentiate into endothelial-like cells. When incubated with VEGF, the expression of VEGFR1, VEGFR2, von Willebrand factor, and CD54 is increased [104, 105]. These VEGF-induced DPSCs exhibited endothelial features and formed capillary-like structures when cultured on a fibrin clot [105]. More recently, a structured dentin-/pulp-like tissue with vasculatures has been created using DPSCs via 3D print technique, suggesting a new direction for customized application for individual design of defect repair [106].

### 2.5. Immunomodulatory Properties of DPSCs

MSCs exhibit some immunomodulatory and anti-inflammatory factors, for example, interleukin-10 (IL-10) [107], hepatocyte growth factor (HGF), [108], transforming growth factor-*β* (TGF-*β*) [109], and prostaglandin E2 [110]. MSCs can act as an immunosuppressive agent by modulating the immune response in inflammatory or autoimmune diseases [111, 112]. DPSCs also have immunomodulatory properties associated with expression of soluble factors that inhibit T cell function. For instance, it has been reported that DPSCs express interleukin-8 (IL-8), interleukin-6 (IL-6), and TGF-*β* via Toll-like receptor (TLR) 4 during neuroinflammation in neurodegenerative diseases [8, 113]. An upregulated expression of TLR4 appeared to increase the expression of IL-8 in DPSCs [114], particularly in SCI crush injury where IL-8 preserves axon integrity and decreases cavitation [115, 116]. DPSCs also express TGF-*β*, HGF, and indoleamine 2,3-dioxygenase (IDO) without prior activation [117, 118] and suppress the proliferation of peripheral blood mononuclear cells and the activation of T cells [119, 120]. Coculture of DPSCs and T cells promoted T cell secretion of human leukocyte antigen-G, vascular adhesion molecule-1, intracellular adhesion molecule-1, IL-6, TGF-*β*, HGF, and IL-10, while it downregulated proinflammatory cytokines such as IL-2, IL-6 receptor, IL-12, IL-17A, and tumor necrosis factor-*α* (TNF-*α*) [121]. It was reported that the proliferation of T cells was inhibited by over 90% when cocultured with DPSCs *in vitro* [8, 122]. In addition, recent studies demonstrated that human and rat DPSCs were able to induce FasL-mediated apoptosis of IL-17 T-helper cells, and rat DPSCs exhibited a very strong ameliorating effect on DSS-induced colitis in mice [123, 124]. The study of Hong et al. reported that DPSCs could modulate immune tolerance by increasing CD4+CD25+FoxP3+ regulatory T cells. The results of the intraperitoneal injection of DPSCs into Balb/c(H-2^d^) mice demonstrated that DPSCs had a meaningful effect on mixed lymphocyte reaction [125]. Studies of Kwack et al. demonstrated that DPSCs could inhibit acute allogeneic immune responses by the release of TGF-*β* as a result of allogeneic stimulation of T lymphocytes and provide a novel insight for the allogeneic transplantation of DPSCs in future clinical use [120]. Recent animal studies conclude that DPSCs could modulate immune tolerance and influence apoptosis via T cells and lymphocytes.

## 3. Dental Pulp Stem Cells (DPSCs) and Central Nervous System Diseases

Traumatic damage to the brain and spinal cord leading to a CNS dysfunction, stroke, Parkinson's disease, Alzheimer's disease, and retinal injury is a common central nervous system disease. The CNS typically has a poor ability to repair and regenerate new neurons because of its limited pool of precursor cells [126, 127], expression of myelin-associated growth inhibitory factors [128], and the inherent propensity of resident glial cells to form scar tissue [129]. At present, it is very difficult to treat CNS diseases with conventional clinical therapies. Some studies have suggested that stem cell treatment may offer a novel therapeutic strategy for CNS disease [127, 130]. The hope is that the application of exogenous stem cells (particularly DPSCs) will lead to both regeneration of new neural precursor cells and their enhanced neuronal and glial differentiation as well as to survival and maintenance of existing neural cells through secretion of trophic factors [29, 30, 40].

### 3.1. DPSCs and SCI

SCI in humans can cause partial or complete loss of motor and sensory function that reduces the quality of an individual's life and leads to an economic burden on society [124, 131]. SCI involves an initial primary tissue disruption (e.g., mechanical damage to nerve cells and blood vessels) and then a secondary injury caused by neuroinflammatory responses (e.g., excitotoxicity, blood-brain barrier disruption, oxidative stress, and apoptosis) [132, 133]. Because of their neural crest lineage, DPSCs have championed as preferred stem cells for SCI therapies supported by growing evidence of DPSCs differentiating into neuron-like and oligodendrocyte-like cells that may promote axonal regeneration and tissue repair after SCI [28, 127, 134, 135]. DPSCs also reduce secondary inflammatory injury, which facilitates axonal regeneration and reduces progressive hemorrhagic necrosis associated with interleukin-1*β* (IL-1*β*), ras homolog gene family member A (RhoA), and sulfonylurea receptor1 (SUR1) expression [136]. When transplanted together with artificial scaffolds such as chitosan, DPSCs promoted motor functional recovery and inhibited cell apoptosis after SCI by secreting BDNF, GDNF, and NT-3 and reducing the expression of active-caspase 3 [8, 137].

### 3.2. DPSCs and Stroke

Stroke is an ischemic cerebrovascular condition that leads to brain damage, long-term disability, and even death [138]. Due to prolonged period of insufficient blood supply and poor oxygen perfusion, damages on affected brain are irreversible. There are unfortunately few effective strategies that can reverse the damage effect on the brain or restore one's function to prestrike level [139]. Recent studies indicate that stem cell therapy may present a novel strategy for stroke treatment due to the multipotency, immunomodulatory, and neuroprotective and angiogenic properties of these cells [140, 141]. Some *in vivo* studies have shown that transplantation of DPSCs into the ischemic areas of middle cerebral artery occlusion (MCAO) in Sprague-Dawley (SD) rats promoted locomotor functional recovery and decreased infarct areas by their differentiation into dopaminergic neurons and secretion of neurotrophic factors [102, 142]. DPSC transplantation into ischemic areas of focal cerebral ischemia in rats led to expression of proangiogenic factors that supported dense capillary formation and renormalization of blood flow [143]. Intracerebral transplantation of DPSCs into regions of focal cerebral ischemia in rodent models promoted forelimb sensory and motor functional recovery at 4 weeks posttreatment [140]. DPSCs also provided cytoprotection for astrocytes by reducing reactive gliosis and preventing free radical and IL-1*β* secretion within *in vitro* ischemic models [86]. Thus, DPSCs may play an immunomodulatory role to promote functional recovery after ischemic stroke.

### 3.3. DPSCs and Parkinson's Disease

Parkinson's disease (PD) is a progressive neurodegenerative condition associated with loss of nigrostriatal dopaminergic (DA) neurons that leads to muscle rigidity, bradykinesia, resting tremor, and postural instability [144]. Stem cell-based therapies hold some promise as a novel strategy for PD treatment [145]. DPSCs can be induced to differentiate into dopamine expressing DA neuron-like cells *in vitro* by using experimental cell induction media [65]. Intrathecal transplantation of DPSCs into the 1-methyl-4-phenyl-1,2,3,6-tetrahydropyridine- (MPTP-) induced old-aged mouse model of PD promoted recovery of behavioral deficits, restored DA functions, and attenuated MPTP-induced damage by reducing the secretion of proinflammatory factors such as IL-1*α*, IL-1*β*, IL6, IL8, and TNF-*α* and by upregulating the expression levels of anti-inflammatory factors such as IL2, IL4, and TNF-*β* [146]. DPSCs also showed neuroimmunomodulatory activity in an *in vitro* model of PD by reducing MPTP-induced deficits associated with reactive oxygen species, DNA damage, and nitric oxide release [146, 147]. DPSCs also promoted survival of DA neurons and enhanced nigrostriatal tract functional recovery in a 6-hydroxydopamine- (6-OHDA-) induced PD rat model by 6 weeks posttransplantation [148]. Some studies have also shown that DPSCs reduced 6-OHDA-induced damage in the *in vitro* model of PD [69, 145]. The clinical use of DPSCs may be a promising approach for treating PD in the future.

### 3.4. DPSCs and Alzheimer's Disease

Alzheimer's disease (AD) is a progressive neurodegenerative condition caused by the loss of neurons, intracellular neurofibrillary tangles, and deposition of insoluble *β*-amyloid peptides in the brain [149, 150]. Clinical symptoms of AD include memory loss, cognitive deficits, and linguistic disorders [150]. Recently, several studies reported that stem cell-based therapies in both *in vitro* and *in vivo* models of AD improved AD-induced pathologies and behavioral deficits [151–153]. DPSCs promoted neuronal repair and regeneration by restoring cytoskeletal structure, protecting microtubule stability, and reducing tau phosphorylation in the okadaic acid- (OA-) induced cellular model of AD [154]. DPSCs can also reduce amyloid beta (A*β*) peptide-induced cytotoxicity and apoptosis in the AD cellular model by secreting higher levels of VEGF, fractalkine, RANTES, fms-related tyrosine kinase 3, and monocyte chemotactic protein 1 [155, 156]. These results suggest that DPSCs are a promising cell source for secretome-based treatment of AD.

### 3.5. DPSCs and Retinal Injury

The retina is a part of the CNS and is composed of photoreceptors, bipolar cells, and retinal ganglion cells (RGCs) [43, 157]. Head injuries can cause traumatic optic neuropathy (TON) while ocular chronic degenerative diseases such as glaucoma lead to the slow loss of RGCs [158]. Retinal and optic nerve injuries have a limited capacity to repair and regenerate because of axon growth inhibitory molecules and reduced production of neurotrophic growth factors [7, 159]. One study reported that DPSC transplantation into the vitreous of optic nerve injury rat model could promote axonal regeneration and RGC survival by a neurotrophin-mediated mechanism [83]. This same study revealed that DPSCs were more beneficial for axonal regeneration than BMSCs because of their higher secretion of neurotrophin factors. A subsequent report showed that intravitreal transplantation of DPSCs in an animal model of glaucoma maintained visual function up to 35 days after treatment by preventing RGC death [160]. Although not assessed *in vivo*, some *in vitro* studies have reported that DPSCs can be induced to differentiate into both RGC-like and photoreceptor cells [161, 162]. Taken together, these results suggest that DPSCs may become an important cell source for stem cell-based therapies in ocular diseases.

## 4. DPSCs and Peripheral Nerve Injury

Peripheral nerve injury caused by traumatic accidents and iatrogenic damage often accompanies physical disability and neuropathic pain. There are many current clinical treatments including direct end-to-end nerve suturing, nerve grafts, and nerve conduits containing growth-stimulatory biomaterials to repair and regenerate injured peripheral nerves [163–165]. Among them, autologous nerve grafting is the gold standard therapy for the long gap of peripheral nerve deficits [166, 167]. However, there are some disadvantages which restrict the clinical use of autografting, such as donor nerve availability and morphometric mismatching [168–171]. With the development of nerve tissue engineering and stem cell-related therapy, various novel nerve conduits in combination with stem cells are providing alternate strategies and approaches for the treatment of peripheral nerve injury [165, 172]. Some studies suggest that DPSC-embedded biomaterial nerve conduits such as polylactic glycolic acid tubes have the ability to promote regeneration of injured facial nerve and to improve functional recovery comparable to that of autografts [173]. Collagen conduits loaded with Schwann-like cells induced from DPSCs *in vitro* have facilitated repair and regeneration of 15 mm sciatic nerve defects [174]. In another report, differentiated DPSCs combined with collagen scaffolds exhibited Schwann cell-related properties and promoted axonal outgrowth and myelination in 2D or 3D culture conditions of an *in vitro* model [78]. Moreover, DPSCs transfected with oligodendrocyte lineage transcription factor 2 differentiated into functional oligodendrocytes *in vitro* and promoted injured peripheral nerve repair and regeneration in a mouse model [175]. DPSCs transplanted into diabetic rats secreted various cytokines that modulated the proportions of M1/M2 macrophages and provided beneficial anti-inflammatory effects in diabetic polyneuropathy [176].

In summary, DPSCs have the capacity to differentiate into Schwann-like and oligodendrocyte-like cells and they secrete neurotrophic factors that provide neuroprotection and modulate the immune response. These cells are poised to become a promising cell source for peripheral nerve injury treatment in the future.

## 5. Conclusions and Future Insights

This review summarizes the neuronal differentiation potential, neuroprotective features, and neurotrophic, angiogenic, and immunomodulatory properties of DPSCs in the pathological and injured nervous system. DPSCs have the biological properties of MSCs and possess a considerable capacity to differentiate into neuron-like cells and secrete neuron-related trophic factors due to their cranial neural crest origin. DPSCs are able to express neuronal markers without preinduced differentiation. Thus, both nondifferentiated and differentiated DPSCs are emerging as new cell sources for the treatment of nervous system deficits associated with SCI, stroke, AD, PD, and long gaps of peripheral nerve injury. DPSCs have several advantages over other exogenous stem cells for nervous system therapies because they are easily harvested without highly invasive surgery, have low immunogenicity, and arise from a neural crest origin that facilitates their neural differentiation. Moreover, after lentiviral transfection with Lin28, Nanog, Oct4, and Sox2 or retroviral transfection with Oct3/4, Sox2, and Klf4, DPSCs can be reprogrammed to generate an embryoid body of iPSCs. The DPSC-derived iPSCs have ability to differentiate into *β*-III tubulin neuron-like cells and tyrosine hydroxylase-positive dopaminergic neuron-like cells and may become another DPSC-related cell sources for the treatment of nervous system diseases in the future.

Because of the vascularization and immunomodulatory properties of DPSCs, these cells can both directly and indirectly stimulate formation of new blood vessels and enhance blood flow to injury sites. In addition to their roles in regeneration and repair of injured neural tissue (Table 1), therapies using DPSCs are emerging as a promising novel strategy for treating other brain conditions and syndromes such as traumatic brain injury, multiple sclerosis, and autism spectrum disorders.

**Figure 3:**
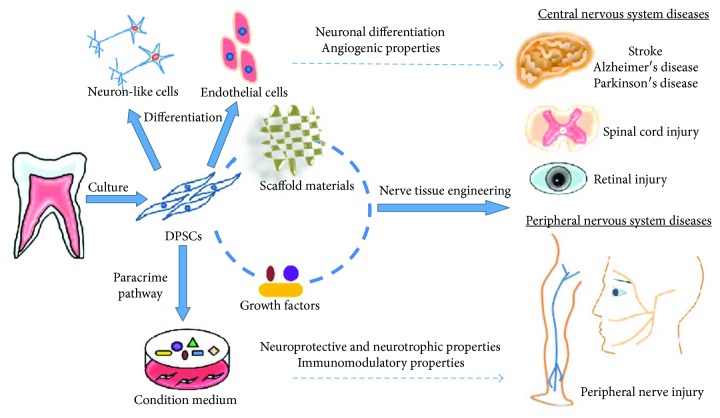
Tissue-engineered constructs of DPSCs, scaffolds, and growth factors and their applications in nervous system diseases. In the constructs, scaffolds can provide biomimetic environments and structural support for cell survival and proliferation. Growth factors can promote neuronal cell proliferation and survival *in vivo* and *in vitro*. DPSCs can enhance neuronal regeneration and repair due to their neuronal differentiation potential and their neurotrophic, neuroprotective, angiogenic, and immunomodulatory properties.

However, despite the functional advantages of using DPSCs for the treatment of nervous system injuries and diseases, there remain significant roadblocks with respect to overcoming the nervous system's seemingly inherent and immutable resistance to regeneration and repair. Nerve tissue engineering approaches are now beginning to adopt combinatorial strategies that involve simultaneous manipulations to cells, growth factors, and scaffolds in order to circumvent the recalcitrant nature of the nervous system (Figure 3). In particular, novel scaffolds such as hydrogels have a 3D porous structure and good cytocompatibility that can be used to provide an *in vivo-*like microenvironment and structural support for cell adhesion, proliferation, and growth. Scaffolds can be designed to embed biological important macromolecules such as bFGF and NGF and to precisely tune their diffusion rate and enzymatic degradation. Seed cells such as DPSCs have beneficial effects on neural regeneration and repair associated with their neural differentiation potential and their neurotrophic, angiogenic, and immunomodulatory properties. Therefore, the spatiotemporal combination of DPSCs, scaffolds, and growth factors provides a promising strategy for treating nervous system-related diseases and injuries in future clinical approaches.

## Figures and Tables

**Table 1:** Examples for the beneficial of DPSCs on the central nervous system (CNS) diseases and the peripheral nervous system (PNS) diseases.

Type of diseases	Author	Differentiated status of DPSCs	Delivery method	Function of DPSCs	References
*The central nervous system (CNS) diseases*					

Spinal cord injury (SCI)	Yamamoto et al.	Undifferentiated	DPSC transplantation	DPSCs inhibited massive SCI-induced apoptosis, preserved neural fibers and myelin, regenerated transected axons, and replaced damaged cells by differentiating into oligodendrocytes	[134]
	Yang et al.	Undifferentiated	DPSCs transplanted with cell pellets	DPSCs reduced inflammatory injury, promoted axonal regeneration, and reduced progressive hemorrhagic necrosis after SCI by inhibiting IL-1*β*, RhoA, and SUR1 expression	[136]
	Zhang et al.	Undifferentiated	DPSCs transplanted with chitosan-scaffold	DPSCs promoted motor functional recovery and inhibited cell apoptosis after SCI through secreting BDNF, GDNF, NT-3 and reducing the expression of active-caspase 3	[137]

Stroke	Song et al.	Undifferentiated	DPSCs cocultured with the conditioned medium *in vitro*	DPSCs conferred superior cytoprotection against cell death by reducing reactive gliosis and suppressing free radical and proinflammatory cytokine expression	[86]
	Song et al.	Undifferentiated	Intravenous DPSC injection	DPSCs reduced the infarct volume of SD rats after middle cerebral artery occlusion (MCAO) due to high angiogenesis and neurogenic differentiation and reduction of reactive gliosis	[87]
	Sugiyama et al.	Dental pulp-derived CD31(−)/CD146(−) side population (SP) stem cells	CD31(−)/CD146(−) SP cells transplantation	DPSCs promoted migration and differentiation of the endogenous neuronal progenitor cells and induced vasculogenesis and ameliorated ischemic brain injury of SD rats after transient middle cerebral artery occlusion (TMCAO)	[102]
	Yang et al.	Dental pulp-derived neuronal stem cells (tNSCs)	tNSC transplantation	Transplanted tNSC promoted function recovery after MCAO because of possessing hypoimmunogenic properties and immune modulation abilities	[142]
	Leong et al.	Undifferentiated	Intracerebral DPSC transplantation	DPSCs enhanced the recovery of poststroke sensorimotor deficits owing to differentiation into astrocytes and mediation through DPSC-dependent paracrine effects	[143]

Parkinson's disease (PD)	Kanafi et al.	Dopaminergic cell-type differentiated	DPSCs were induced *in vitro*	DPSCs showed efficient propensity towards functional dopaminergic cell type	[65]
	Chun et al.	Dopaminergic neurons differentiated	DPSCs were treated with the dopaminergic neuron differentiation kit *in vitro*	DPSCs could differentiate into dopaminergic neural cells under experimental cell differentiation conditions	[69]
	Gnanasegaran et al.	Undifferentiated	Intrathecal DPSC transplantation into a mouse model of PD *in vitro*	DPSCs could treat the PD by regulating inflammatory mediators such as reducing the secretions of proinflammatory factors (IL-1*α*, IL-1*β*, IL6, IL8, and TNF-*α*) and upregulating the expression levels of anti-inflammatory factors (IL2, IL4, and TNF-*β*)	[146]
	Gnanasegaran et al.	DAergic-like cells differentiated	DPSCs were cultured in a system which consists of neuron and microglia *in vitro*	DPSCs were shown to have immunomodulatory capacities to reduce 1-methyl-4-phenyl-1,2,3,6-tetrahydropyridine- (MPTP-) induced deficits such as reactive oxygen species, DNA damages, and nitric oxide release	[147]

Alzheimer's disease (AD)	Wang et al.	Undifferentiated	DPSCs cocultured with okadaic acid- (OA-) induced cellular model of AD *in vitro*	DPSC-treated cells had the morphology of restored neurons, elongated dendrites, densely arranged microfilaments, and thickened microtubular fibrils	[154]
	Ahmed et al.	Undifferentiated	DPSCs cocultured with amyloid beta (A*β*) peptide-induced cellular model of AD *in vitro*	DPSCs secreted and produced numerous vascular endothelial growth factor (VEGF), fractalkine, RANTES, fms-related tyrosine kinase 3 (FLT-3), and monocyte chemotactic protein 1 (MCP-1)	[155]

Retinal injury	Mead et al.	Undifferentiated	Intravitreal DPSC transplantation	DPSCs produced and secreted lots of neurotrophins in order to promote neuritogenesis/axogenesis of retinal cells	[83]
	Mead et al.	Undifferentiated	Intravitreal DPSC transplantation	DPSC provided protection from retinal ganglion cell (RGC) loss and retinal nerve fiber layer thickness (RNFL) thinning and preserved RGC function	[160]
	Bray et al.	Undifferentiated	DPSCs cocultured with the conditioned media which were obtained from organotypic explants from damaged rat retinas *in vitro*	DPSCs had ability to promote neurodifferentiation and expression of retinal neuronal markers in order to cure the rat retinas	[161]

*The peripheral nervous system (PNS) diseases*					

Facial nerve defect	Sasaki et al.	Undifferentiated	DPSCs transplanted with poly-dl-lactide-coglycolide (PLGA) and collagen gel	DPSCs promoted the axon regeneration and myelinated nerve formation	[173]
Sciatic nerve defect	Sanen et al.	Schwann cell-type differentiated	DPSCs transplanted with NeuraWrap™ conduits	DPSCs promoted in growing neurites, myelinated nerve, and newly blood vessel formation and survival	[174]
Sciatic nerve defect	Askari et al.	Oligodendrocyte progenitor cell- (OPC-) type differentiated	DPSC-induced OPC transplantation	DPSCs could be differentiated into functional oligodendrocytes	[175]
Sciatic nerve defect	Omi et al.	Undifferentiated	DPSC transplantation	DPSCs increased the gene expression of interleukin-10 and promoted macrophages polarization towards anti-inflammatory M2 phenotypes	[176]
